# Beliefs and Norms Influencing Initiation and Sustenance of Exclusive Breastfeeding: Experiences of Mothers in Primary Health Care Facilities in Ermelo, South Africa

**DOI:** 10.3390/ijerph20021513

**Published:** 2023-01-13

**Authors:** Perpetua Modjadji, Ethel Sekori Seabela, Busisiwe Ntuli, Sphiwe Madiba

**Affiliations:** 1 Non-Communicable Diseases Research Unit, South African Medical Research Council, Cape Town 7505, South Africa; 2 Department of Public Health, School of Health Care Sciences, Sefako Makgatho Health Sciences University, 1 Molotlegi Street, Ga-Rankuwa, Pretoria 0208, South Africa; 3 Faculty of Health Sciences, University of Limpopo, Polokwane 0700, South Africa

**Keywords:** views, lived experience, exclusive breastfeeding, mothers, primary health facilities, Ermelo, South Africa

## Abstract

Exclusive breastfeeding (EBF) is not a norm in many communities in South Africa despite the World Health Organizations’ recommendations for EBF in the first six months of infant’s life. Thus, South Africa continues to observe suboptimal and poor practices of EBF. The purpose of the study was to explore the experiences of mothers who are HIV-positive and negative on EBF and examine the extent to which initiation and sustenance of EBF is influenced by cultural beliefs, societal norms, and family norms and practices in Mpumalanga Province. Three focus group discussions and twelve in-depth interviews were conducted among thirty mothers who were purposively selected during their visits to the facilities for childcare services. Interviews were audiotaped, transcribed verbatim, and transcripts were analysed through thematic analysis using NVivo version 10. Mothers were aged between 18 and 42 years, most were unemployed and were living in poor sociodemographic backgrounds in extended family households. We found evidence of factors that influence the decision to EBF and mix feed infants among mothers. Traditional and cultural beliefs and norms that exist within their communities informed decisions mothers took to EBF. These beliefs existed alongside mothers’ opinions on breastfeeding (BF) and HIV infection, as well as the fears of harming the baby through HIV infection, leading to early cessation of BF. Mothers were also advised by family members, friends, and even some healthcare workers to use traditional medicines while BF. The association of EBF with sagging breasts and weight loss as well as discomfort with public BF are personal beliefs that influenced initiation and early cessation of EBF. Breastfeeding messages ought to be context specific to improve the knowledge, understanding, acceptance and practice of EBF among HIV-positive and negative mothers. Culturally appropriate counselling messages that address the known cultural practices of the populations affected are essential to changing the beliefs and norms of the communities including extended families of EBF mothers.

## 1. Introduction

The World Health Organization (WHO) defines exclusive breastfeeding (EBF) as feeding infants only breast milk, either directly from the breast or expressed, with no addition of any liquid or solids apart from drops or syrups consisting of vitamins, mineral supplements or medicine, and nothing else [1]. EBF is followed by the introduction of nutritious and safe complementary foods with continued breastfeeding (BF) for at least two years [2]. WHO guidelines on infant feeding have drastically evolved from 2001 to 2016, with the timeline for BF currently extended to at least two years [2]. One of the global nutrition targets of WHO and United Nations International Children’s Emergency Fund (UNICEF) [3] is to increases the rate of EBF in the first six months up to at least 50% at country-level by 2025, which will contribute towards the achievement of the Sustainable Development Goals (SDGs) [4]. UNICEF asserts that EBF improves nutrition (SDG 2), prevents child mortality, decreases the risk of non-communicable diseases (NCDs) (SDG 3), and supports cognitive development and education (SDG 4) [5]. In 2011, South Africa changed the infant feeding policy through the Tshwane Declaration to EBF for all women regardless of human immunodeficiency virus (HIV) status [6,7]. The declaration led to a shift in the counselling given to HIV-positive women, and it also marked an end to free distribution of infant formula for women with HIV at public health facilities [6,8].

In 2019, only 41% of infants under six months of age were exclusively breastfed, worldwide [9]. South Africa’s average EBF rate has increased to 32% [10] from a 7% rate that was reported in 1998 [11]. Most women (77–90%) start BF within an hour after birth [12], as an acceptable method of infant feeding, even in the African culture [13]. At an individual level, maternal education, comfort in BF, social class, desires, and attitudes of mothers regarding BF, influence the decision to initiate and continue to breastfeed [12,13,14]. While initiation of BF is high in South Africa, low rates of EBF have been reported [15]. Early cessation of BF and mixed feeding are common among South African mothers, including addition of other liquids and complementary feeds in the first six months of the infant’s life being a norm [8,10]. There is consensus among researchers that EBF is not consistent with African cultural values and traditions [13]. Thus, EBF has been consistently low in sub-Sahara Africa, as many mothers are not able to practice EBF for the first six months of the infant’s life [13]. A systematic review of 195 countries reported that only 37% of infants under six months of age in Africa were exclusively breastfed in 2017 [16].

Cultural factors are primary inhibitors of EBF in many societies, including South Africa [17,18]. Of concern to public health is that healthcare workers (HCWs) in high prevalence settings also believe in these cultural practices and often provide conflicting messages, which are confusing to mothers [13,19]. African traditions encourage the use of medicinal herbs to infants in keeping with traditional practices, and mothers are obliged to follow these traditions [19,20]. Additionally, the support from partners, family, and HCWs plays an important role in influencing a mother to exclusively breastfeed [21]. The heads of the household, grandmothers, mothers in law, and other extended family members have overwhelming influence on decision-making about EBF for mothers [13,14,18,22,23]. Furthermore, generational infant feeding practices are passed on to young mothers who are largely influenced by these practices [13,17,24]. For example, cultural and family norms that support the early introduction of food and liquid conflict with EBF [17,18,23,25].

Other factors that influence mothers’ decisions to exclusively breastfeed for the first six months include BF in the workplace, as well as teenage mothers leaving their babies to go back to school [18,26,27]. Confusion about the risk of HIV transmission and BF remain an inhibitor to EBF in South Africa, and early in the HIV epidemic women with HIV were recommended not to breastfeed [28]. The frequent changes to the infant feeding guidelines for mothers with HIV have resulted in contradictory infant feeding messages [28]. Often the advice or counselling from HCWs is not supportive of EBF [19,27]. Thus, fear of HIV transmission to their infants continues to influence EBF even though antiretroviral therapy (ART) has made BF for HIV-positive women safe [27,29]. Most mothers with HIV do not initiate BF or either they do so for a short duration [15,18].

As stated, EBF in the first six months is not a norm in many communities, including South Africa and the proportion of exclusively breastfed children remains below recommended global targets for those communities [29,30], where high child deaths attributed to suboptimal BF occur [30]. There is an indication that evidence-based knowledge on the benefits of EBF, as well as the concerted efforts through policy reform, have not translated into practice in South Africa and other African countries. As a result, these countries continue to observe suboptimal EBF in the first six months of the infant’s life [18,31,32,33]. Evidence from a systematic review of 195 countries and the mapping of 49 African countries found that most countries will not meet the global nutrition target of 50% EBF prevalence in the first six months by 2025 [16,17].

Despite this target [34], research on infant feeding practices in South Africa tends to focus on women with HIV [18], whereas the global targets include both HIV positive and negative women. Research suggests that low EBF rates are attributed to historical infant feeding policy that was driven for the needs of HIV-positive women. The focus on EBF for HIV-positive women might have influenced HIV-negative mothers BF practices [15,23]. There is no evidence that EBF rates significantly increased after the Tshwane Declaration in South Africa in 2011 [15]. Furthermore, there is lack of data on the aspects of EBF that affect its translation to practice in South Africa and other settings in the African content [13].

Therefore, the purpose of the study was to explore HIV-positive and negative mothers’ experiences on EBF during the first six months of the infant’s life and examine the extent to which initiation and sustenance of EBF is influenced by cultural beliefs and societal and family norms in Mpumalanga Province, South Africa. Although a systematic review by Nieuwoudt and colleagues [18] has reported substantial BF research conducted in South Africa between 1980 and 2018, few studies have been conducted in four provinces of South Africa’s nine provinces, including Mpumalanga Province. More work is required to explore some aspects of EBF that conflict with African cultural and traditional beliefs and practices and how the conflict affects the adoption of EBF in African context [13]. Moreover, cultural beliefs contributing to EBF practices, varies across communities in South Africa as in other African countries [17]. For South Africa to reach the global nutrition goal of 50% EBF in the first six months by 2025 more socio-cultural and contextual processes should be explored to support optimal EBF. Oyelana and colleagues [13] argue that EBF recommendations should be modified to accommodate African traditional values system. Therefore, there is need for policy makers to develop strategies and intervention programmes that acknowledge and respect the sources of socio-cultural beliefs without endangering the infant’s health [18].

## 2. Materials and Methods

### 2.1. Study Design, and the Conceptual Frameworks

An explorative descriptive study design using a qualitative research approach was employed to study mothers’ experiences on EBF during the first six months of the infant’s life and to examine the extent to which initiation and sustenance of EBF is influenced by cultural beliefs and societal and family norms. The study adhered to the Consolidated Criteria for Reporting Qualitative Research (COREQ): a 32-item checklist for interviews and focus groups [35]. The study was conducted from May 2019 to October 2019.

This study is anchored on the the conceptual framework of factors affecting BF, which proposes that the individual, group, and society are the three levels influencing EBF [36]. This framework guided data collection, including development of field guides, and analyses. According to Hector et al. [36], influences from the individual level relate directly to the mother, infant, and the mother–infant dyad. The focus is on the mother’s intention to breastfeed, including other aspects, such as parenting experience, the birth experience, health and risk status of mothers and infants, and the nature of early interaction between mother and infant. On the group level, influences originate from the attributes of the environment in which mothers and infants find themselves, as well as enablers to breastfeed, such as the health facility, infant feeding counselling, and providing professional support with BF technique difficulties. This is in addition to the home where physical and social factors such as size of household, parity, and partner attitudes and support, affect the time, and determination that mothers have for breastfeeding. The community environment also signals the extent to which BF is recognized as a norm and reinforced by facilities and policies in public places. The third level, which is the societal level, influences the acceptability and expectations about BF and provides the background or the context in which mothers’ feeding practices occur. These include cultural norms regarding BF, child feeding, and parenting, the extent to which men’s social role includes support for BF mothers; the extent to which exposing breasts for feeding is complicated by cultural norms regarding sexuality, as well as the economic importance of products such as breastmilk substitutes and complementary foods in the food system [36].

### 2.2. Study Setting and Population

The study was conducted in Ermelo, a small town surrounded by several townships, and farms, situated in Gert Sibande District, one of the three districts of Mpumalanga Province of South Africa. Ermelo has an approximated total population of 164,608, and it is serviced by three primary health care facilities (PHC) and a regional hospital. Data were collected in the three PHC facilities; two are in the township (i.e., peri-urban) and one is in a town (i.e., urban). Although there are a variety of different tribes and languages, most of the population in this area are black and speak IsiZulu.

Mothers of children under the age of two years, who visited the facilities for childcare services were selected using purposive sampling to participate in the study. In purposive sampling participants are selected because they can provide in depth and meaningful information about the topic of the study [37]. About 30 to 50 mothers attended childcare services daily from Monday to Friday per week. During the visits to the facilities, mothers were informed about the study by the principal researcher who is a registered dietician. Mothers were eligible to participate if they were 18 years old with a child aged under two years, had ever breastfed, or were BF at the time of the study, and were able to provide a written informed consent. Potential participants were recruited while in the queues for services, and those who met the inclusion criteria were identified and approached with the help of the nursing staff at the respective facilities. The researcher and the research assistant explained the purpose of the study in detail and those who volunteered to participate were requested to see the researcher for arrangements to form focus group discussions (FGDs) or individual in-depth-interviews (IDIs).

### 2.3. Data Collection, Tools, and Procedure

A semi-structured interview guide, informed by the determinants of EBF conceptual framework [36], was developed by the research team. The interview guide was developed in English and translated into IsiZulu. The guide consisted of open-ended questions which addressed questions on initiation of EBF, experiences of EBF, the influences of significant others on the decision to EBF, the influences of significance others to introduce other liquids and solid foods, BF and HIV, and infant feeding practices in general. During interviews follow-up questions and probes were asked to seek clarity or further explore responses given by the mothers. The study used a combination of IDIs and FGDs to produce in-depth views of mothers on initiation and sustenance of EBF for the first six months of the infant’s life.

Thirty (30) mothers were sampled in this study, and 18 participated in three FGDs consisting of six members per group, while 12 participated in IDIs, and sample size was determined by saturation. Data saturation occurred when no new information on the topic could be obtained both on a question level and entirely from the tool. Data collection and analysis ran concurrently for 24 weeks. Data saturation was reached when further coding did not yield new data, as we kept getting repeated information and no new codes emerged from the data. Consistent with the literature, we found that three FGDs including the 12 IDIs were sufficient to achieve data saturation [38,39,40]. The IDIs lasted for 30 to 35 min, while FGDs took approximately one hour. All interviews were audiotaped with the consent of the participants, which was obtained before the interviews took place. During FGDs, moderation was conducted by the researcher, while handwritten notes were taken by the research assistant. Both FGDs and IDIs were conducted in a private room in the facilities on the same day without interfering with the daily running of the facilities. At the end of each FGD and IDI, sociodemographic data on personal information (i.e., age, marital status, education level, employment status, household family income), as well as obstetric history (i.e., parity, number of pregnancies, mode of delivery of the current baby, baby age and gender, and mother and child HIV status), were collected using a short demographic tool. Refreshments were offered to mothers at the end of interviews.

### 2.4. Data Management and Analysis

Qualitative thematic analysis was the approach used to analyze the data following steps outlined by Braun and Clarke [41] An experienced transcriptionist transcribed verbatim all the interviews from the audio files that used the language of the participants to best represent the dynamic nature of the living conversation. Transcripts were translated into English and were reviewed by the researcher and other authors to ensure their accuracy and that no meaning was lost between the transcription and the translation. The six steps for qualitative data analysis procedures were followed. First, the transcripts were read repeatedly by all authors to familiarize and immerse themselves with the data. This was the first step of data analysis, which yielded several initial codes using manual coding. Second, the initial codes generated from reading transcripts (two for each author) were used to develop a codebook. The authors engaged in a rigorous process to define and name codes, emerging themes, and subthemes. Once the initial codebook had been developed, all the transcripts from the FGDs and IDIs were uploaded in NVivo version 10 [41], a qualitative data analysis software for application of coding to all the transcripts. During the application of codes, the authors reviewed, refined, and combined identified themes and subthemes and finalized themes and subthemes to produce the report. Findings are presented in themes and quotations that reflected mothers’ decision making regarding EBF.

Various strategies were engaged to ensure trustworthiness. We made use of a good digital recorder to aid verbatim transcription, used the local language to conduct the interviews, recorded extensive field and interview notes, and used NVivo version 10 data analysis software for data coding. In addition, peer debriefing sessions were held after each FGD and IDI, and data and investigator triangulation were employed. Records showing data collection processes, analyses, and findings were kept as an audit trail [42]. Bracketing was maintained to reduce inherent biases, as described by Fischer [43]. The principal researcher set aside all preconceived ideas about the EBF during data collection and analysis to ensure credibility of the data analysis and interpretation. In a similar manner, SM and BN were previously engaged with research on BF used bracketing throughout the data analysis and interpretation [44].

### 2.5. Ethical Considerations

This study received ethical clearance from Sefako Makgatho Health Sciences University Research and Ethics Committee (SMUREC) (SMUREC/H/23/2019: PG). Permissions were obtained from the Department of Health, Mpumalanga Province in South Africa (Approval reference number: MP_201905_004), and from the managers of the selected facilities in Ermelo to conduct the study. Written informed consent was obtained from all the mothers who participated in the study, prior to the interviews. Mothers were informed that participation was voluntary, and pseudonyms were used to maintained confidentiality.

## 3. Results

### 3.1. Sociodemographic Status and Obstetric History

Table 1 shows the characteristics (frequency (*n*), and the percentage (%) of mothers. The sample included mothers attending childcare services. Mean age for mothers was 27 years (SD = ±6), ranging from 18 to 42 years, 25 mothers were single, and 22 were unemployed with a household family income of R2000 ($122.92) per month. Only two mothers had obtained tertiary education, 10 had primary education, while 18 had completed the 12th Grade. As to the obstetric history, 20 of the mothers reported unplanned pregnancy, 26 had attended ante natal care (ANC) late, while four mothers never attended, nine had caesarean section, while 14 of the mothers had either three or more children (Table 1).

### 3.2. Characteristics of Children

Table 2 shows the frequency (*n*) and percentage (%) of children’s characteristics. The sample consisted of more boys (20 out of 30 (67%) than girls 10 out of 30 (33%), 70% (21 out of 30) of them were below the age of one year, with a mean age of nine months (SD = ±7), and 23 out of 30 (77%) were born last. All the children were reported to be HIV negative at the time of the study.

### 3.3. Emergent Themes

Three main themes emerged from the study, which are (1) benefits of BF with six subthemes, (2) fears and distress of EBF with three subthemes, and (3) cultural beliefs and EBF with four subthemes, (Table 3).

#### 3.3.1. Child and Maternal Benefits of BF

The theme benefits of BF describe the mothers’ narratives about the importance of BF. Awareness of the potential benefits of BF and BF knowledge influenced the decision to breastfeed for women. Although the focus of the study was EBF, during the interviews, the understanding of mothers on the concept of EBF was linked to their knowledge and practice of BF. The mothers valued the importance of BF and were knowledgeable about the benefits of BF. They mentioned several benefits of BF, which included cognitive and physical development, protection against infection, diseases, or childhood illnesses. The mothers further showed that they were knowledgeable about the psychosocial aspects of BF, such as the promotion of mother and child bonding. The economical aspect of BF was also highlighted by the mothers, as they indicated that breastmilk is always readily available and economical particularly for most mothers who are employed. However, the narratives of the mothers did not mention or indicate what EBF offers to the child. The following excerpts further describe what the mothers had to say about BF.

▪ BF promotes child growth and development.

A key benefit that was commonly cited was that BF promotes physical growth and cognitive development. They perceived their children to grow well because they were breastfed. This is what mothers said,

*“I could even see that when the child was put on the weighing scale, the child was picking up weight because I checked the clinic card. Whenever I bring the child up for weighing, I would look at it each month and could see increases at that time I was only breastfeeding that one. I could see that the child is growing)”.*

(FGD 3, Participant 4)

*“Breastfeeding is important, it make the child to grow well, be healthy, and to have a good weight”.*

(IDI. Participant 3)

▪ BF offers protection against childhood illnesses

The mothers’ narratives indicated that they were knowledgeable about the difference between breast-fed and formula fed babies. They acknowledged that breast-fed babies are healthier than those who are formula fed and highlighted BF as the healthier choice of infant feeding. They perceived that because they breastfed their babies, they were healthy and did not have any health problems. Their narratives indicated that the health benefits of BF influenced the decision to breastfeed.

*“I can say that most mothers breastfeed their children because breastfeeding children don’t get sick often, they grow well, actually they only come to the clinic for weight monitoring and not because the child is sick”.*

(IDI, Participant 7)

*The practice of breastfeeding is good, because it protects the infants from illness..., and yes breastfeeding is good”.*

(FGD 2, Participant 1)

*“Ok, with this breast milk, my child did not have this funny diarrhoea. She was ok and all that, but the minute she stopped breast milk and when I gave her formula, she had diarrhoea”.*

(IDI, Participant 5)

▪ BF promotes mother-baby bonding

Consistent with the global literature, for most of the mothers, BF had a significant influence on mother-baby bonding. The belief that BF increases the bonding between the mother and her baby influenced the decision to initiate and sustain BF among the mothers. This is what they had to said,

*“I had that thing that I want my child and I to bond and that she can feel that I am her mother, so when I put her on the breast, she could feel that I am her mother”. I enjoyed breastfeeding, it pained me that I had to stop you see, I enjoy that there is this connection we have, when I breastfeed my child, it is just nice, like looking at the child while breastfeeding it’s just yes, it is nice”.*

(IDI, Participant 9)

*“I am happy about breastfeeding my child because even the child looks at, touches me, and knows that this one is my mother. She/he feels the love from me because during breastfeeding I brush the child”.*

(IDI, Participant 2)

*“It is not something that you are forced to do; it is out of love of it, yes. I think it creates a bond between you and the child when you breastfeed”.*

(IDI, Participant 8)

*“I just feel that breastfeeding is important, it is important it keeps the bond between the mother and the child, and your child will be depending on the mother….*

(IDI, Participant 10)

▪ BF is economical and convenient

The mothers appreciated the fact that breastmilk is available at any time, and it is not expensive given the cost of purchasing formula feeding. Most of the mothers were unemployed and reported that they would not be able to afford and sustain formula feeding.

*“If you are not working like me, you won’t be stressing out about milk formula running out after two months or two weeks that you don’t have the money to buy it and all that there won’t be that”.*

(IDI, Participant 6)

▪ BF promotes maternal mental wellbeing

Besides citing how BF benefits babies, the mothers perceived BF as important for their mental health, their narrative revealed that they experience bonding with their babies during BF. This brings about happiness which is important for the wellbeing of mother and child.

*“I was happy that I could communicate with the child, we would look at each other in the eyes during breastfeeding, I could see that that child is all good and not sick, it means it is nice to breastfeed”.*

(IDI, Participant 7)

*“I feel happy always, because breastfeeding is in my blood, I love it, I enjoy it, I feel really good about it even when I am breastfeeding my mind is just free, I am not thinking of problems, my concentration would be on the child”.*

(IDI, Participant 6)

▪ Child spacing

Breastfeeding in many societies and cultures has often been regarded and used to lengthen the time between births of successive children. Delayed return to fertility is one of the cited benefits of BF to the mothers. One mother has this to say about this belief;

*“Breastfeeding can delay the next pregnancy”.*

(IDI, Participant 9)

#### 3.3.2. Fears and Distress of the Effect of EBF

While mothers appreciated the benefits of BF, at the same time, those who were infected with HIV had fears of having to exclusively breastfeed. Despite evidence of receiving counselling and education on the low risk of MTCH through EBF, they did not trust the information they received from nurses. What was discussed during infant feeding counselling and the beliefs about HIV transmission posed challenges for mothers to exclusive breastfeed. As a result, some of them stopped BF and introduced milk formula. The excerpts below describe the narrated fears:

▪ Fears of harming the baby.

For mothers to make an informed decision to EBF they need to understand the concept of EBF, the protection offered by HIV medication, and low risk of the mother infecting her baby with HIV through breast milk during counselling. Whilst mothers certainly received counseling, some of them remained doubtful and concerned that their babies might be infected during breastfeeding.

*“I did not trust what the nurses said. Nurses said the child would not be infected because they have educated us that HIV can be transmitted through blood. I stopped breastfeeding because I was scared that the child would be infected with HIV”. She further said “I stopped breastfeeding the child because I was scared, my breast was cracked, it looked like it was cut, like blood was coming out.*

(IDI, Participant 9)

▪ Belief that the baby is not getting enough milk.

The perceived belief that breastmilk is inadequate led to mothers believing that they were harming their babies. This belief influenced the early introduction of solids and other liquids to alleviate the baby’s hunger, as mothers perceived persistent crying of the baby to be an indication of hunger. The belief that breastmilk is inadequate was also influenced by the opinions and pressures from families to introduce solids early in the life of the infants.

*“Breast milk is water and water does not satisfy…, it is just that it is coloured, and is milk. Alone it will never satisfy the baby. You will always need to also feed the child solid food on a side.”*

(FGD 1, Participant 2)

*“Because these children do not get satisfied with breast milk and the more the child cries the more you become stressed and when you are stressed yet on the other hand you are breastfeeding, you just end up causing the child some tension”, said one mother.*

(IDI, Participant 4)

*Another mother said, “At home, my mother decided that it was better that I give my child food so that we can see whether he is crying or if the baby wanted food”.*

(FGD 3, Participant 5)

▪ Fears of effect of EBF on body image.

Some mothers indicated that because of sagging breasts due to BF they would not be able to breastfeed for the stipulated duration. Data revealed that some mother’s personal fears were evoked by anticipated consequences of BF on losing their partners, change in body image, and maternal stress and tension caused on the baby through breastfeeding.

*“What I can say is that I won’t breast pump again because I think it’s making my breasts to sag, because breast pumping every day is like breastfeeding, you do know right that when women breastfeed, the breasts sag, on top of that you still breast pump”.*

(FGD 3, Participant 4)

*Another mother said, “My breasts are no longer the same size, I breastfeed on only one breast and my body shape is no longer the same”.*

(FGD 1, Participant 3)

While in many affluent societies, women are more concerned about weight gain during pregnancy and BF, the mothers in this study were concerned about the loss of weight associated with EBF. This is particularly important in the context of HIV prevalence and stigma associated with severe weight loss for HIV-positive people. One mother said.

*“It was not comfortable, losing weight and looking thin. But due to exclusive breastfeeding a person looks at you as if you are sick with HIV”.*

(IDI, Participant 11)

The narratives with the mothers revealed that BF has the potential to affect the relationship with their partners. Young mothers in particular reported fears of losing a partner.

*“Isn’t it that some boyfriends do not like breastfeeding mothers. Let us say maybe he says let us meet and you then come with your leaking breast, they do not like that, some do not like that, some would even end the relationship because of that”.*

(IDI, Participant 7)

#### 3.3.3. Cultural Beliefs Influencing EBF

The belief systems of individuals, family, significant others, and society in general play an important role in the decision making of mothers about infant feeding practices, including EBF. The family and community infant feeding culture and practices of BF mothers is important in influencing BF practices of mothers. For some of the mothers, BF comes naturally, and they reported that they breastfed their babies to follow in the steps of their mothers who breastfed them and their siblings. There are many other beliefs that influenced infant feeding choices for mothers, which were rooted in cultural norms and tradition, while some were personal.

▪ Personal beliefs.

*“I think it is a personal decision to decide whether it is good to give breast milk only or if you want to give both.” (IDI, Participant 11). Another one said, “I think it’s a belief thing…, older people believe in cultural practices…, it is a belief thing, because they believe that when you breastfeed and give solids food at the same time, the child would be well, healthy, and grow well”.*

(FGD 3, Participant 6)

▪ Cultural and societal norms

The mothers’ narratives indicated that while some of them had their own individual beliefs and attitudes about EBF, cultural norms, and traditional beliefs, played an important role in influencing them to initiate and sustain EBF, to opt for formula feeding, or mix feed. As indicated, some of the mothers who initiated BF did so to follow the family culture of BF.

*One mother said, “As for me yes I choose to breastfeed because my mother breastfed me, so I just want to follow the way of the breast and breastfeed my child”.*

(IDI, Participant 10)

▪ BF in public spaces

Traditionally, BF in many African societies has no boundaries, women could breastfeed anywhere at any time without shame. The societal norms have changed however, and public BF has emerged as one of the problems for mothers who want to exclusively breastfeed. The expectation and norms are that women cover the breast with a blanket when they breastfeed in public places. The mothers in this study seem to support the view that women should not breastfeed in public places. They stated that covering the breast with a blanket when BF in public places was sign of showing self-respect. This is what mothers said.

*“No, I do not have a problem because I cover my breast. They have taught us that each time when you are breastfeeding you must cover the breast because the breast must be respected; yes, the public must not see your breast, so I do cover up”*

(IDI, Participant 6)

*“There was a time I breastfed my baby in a train, the baby pressed and squeezed breast milk and it spread out to the other guy, some people would not like it. Many people will not enjoy watching our sagged breasts, some say it is disgusting, so I think having a private room where you can breastfeed your baby is nice”.*

(IDI, Participant 10)

Although covering the breast was not an expectation when BF at home, the data suggest that women in this community believe and support covering the breast during feeding. This is what one mother said

*“At home we don’t have those beliefs that maybe you don’t breastfeed the child in front of a man, or you don’t take out the breast, things like that, isn’t it that when I breastfeed her, I can take a blanket to cover”.*

(IDI, Participant 5)

▪ Traditional beliefs influencing EBF.

Mothers in this study reported traditional practices that affect their efforts to exclusively breastfeed their infants for the first six months. In most African societies, performing traditional rituals on babies to protect them from evil spirits is a norm. The mothers reported that babies under six months are given traditional medicines: [imbiza] and other concoctions for the treatment of *ibala* [maroon birth mark at the back of the head and neck], as well as colic and *inyoni* [described as loose stools more similar to diarrhea].

*One mother said, “just after I was discharged from the hospital maybe after two weeks of discharge when the child cried too much and would not stop crying, I took the child to the healer who made a cut [razor cuts on ibala, birth mark] and then gave me imbiza [traditional medicine] for the child to drink”.*

(IDI, Participant 9)

Another mother said,

*“When the child is passing out loose stools, they call it inyoni, so they make razor cut around the umbilicus and put the traditional medicine and also the child is given traditional medicine to drink”.*

(FGD 2, Participant 3)

Some mothers did not believe in giving their babies traditional medicines for the treatment of colic. They used over the counter medications such as Lennon Products for infantile colic. One mother said,

*“I don’t give anything traditional; I give only Western medicine for such things as in case the child might absorb the bad spirits, I use the Western ones like stapes drupels you see” (IDI, Participant 6). The other mother said, “As for us at home, we don’t use cultural medicine, so actually when you get a baby, you must just breastfeed, otherwise you just buy medicines like Phillips Gripe water, if the baby is having a troubled tummy, Bascopan, you give those”.*

(FGD 1, Participant 4)

## 4. Discussion

In this study, mothers were interviewed to explore their experiences on EBF during the first six months of the infant’s life and examine the cultural and traditional beliefs that influence the decision to EBF. We found evidence of factors that influence the decision to EBF or mix feeding their infants in the first six months among HIV-positive and negative mothers. The traditional and cultural beliefs and norms that exist within the communities where mothers live informed the infant feeding practices and decisions they take. Consistent with women in studies in South Africa and other African societies [20,29,45], the social and cultural contexts of women played an important role in their initiation and sustenance of EBF.

The use of herbal concoctions as medicines to treat infantile colic and prevent bad spirits reported by mothers in the current study have been reported among mothers in other countries [19,46,47]. The traditional concoctions are introduced before the completion of the EBF period of six months, thereby interfering with EBF. The use of traditional herbal medicine is apparent in many African population groups [48]. Ingestion of traditional medicine has adverse effects and toxicity due to the differences in physiology, immature metabolic enzyme systems, and dose per body weight for infants [49]. Mothers are advised by their mothers, their grandmothers, other family members, friends, and even some HCWs to use traditional medicines while practicing EBF [50]. Mostly, mothers, mothers-in-law, and grandmothers of the BF mother are the custodians of these norms, beliefs, and systems. This makes the practice of EBF difficult because mothers often feel compelled to submit to the dictates of their culture. Other studies reported similar practices [47,50]. Conforming to cultural norms and traditional beliefs contradicts the guidance of HCWs informed by scientific evidence on EBF practices.

The HIV pandemic has significantly disfigured the face of breastfeeding throughout the world [47]. South Africa has one of the highest rates of HIV (19.2%) [51], thus the country is still faced with suboptimal EBF outcomes [18,31,33]. Furthermore, BF by mothers with HIV remains a complicated dilemma for HCWs. In the current study, mothers with HIV indicated not trusting HCWs when it comes to messages regarding the safety of EBF. As such, the fear of infecting their babies with HIV through BF led to early cessation of breastfeeding and introduction on solids and other liquids. While the fear of infecting their babies with HIV is an important concern for mothers with HIV in this study and others [29], counselling messages need to address the fears and assure mothers of the effectiveness of ART in preventing transmission of HIV to the baby, but also stress the importance of adherence to treatment.

Seemingly, the changing policies on BF might have had a negative impact on messages that women receive during infant feeding counselling. There is substantive evidence that women receive mixed and often confusing messages on EBF and HIV, putting them in a difficult position of whether to breastfeed or not. It is important that information communicated to mothers on BF is consistent and independent of the HCWs’ personal opinions, values, and convictions. While improving knowledge, understanding, acceptance, and practice of EBF among mothers with HIV is necessary, as well as the support from family members to ensure that mothers develop a positive attitude towards EBF. South Africa is not on a positive trajectory to meet SGDs and infant mortality and morbidity targets [18]. Therefore, it is imperative that the promotion of EBF takes center stage in infant feeding health promotion and education messages that women receive during ANC and early postpartum period.

We found that when mothers’ initiation of EBF is regarded as a personal practice, this interfered with BF in general and EBF specifically. Some mothers in this study associated BF with sagging breasts and weight loss, which they perceived as unattractive body image. The body image concern reported is consistent with other researchers [52]. The fear of sagging breasts among mothers influenced the initiation of BF. While, for BF mothers, body image concerns lead to early cessation of EBF. The effect of body image concern and shortened BF duration have been reported in previous studies [53,54]. Studies in African countries, such as Kenya [55] and Ghana [56], have reported that young mothers are always concerned about the perceived effect of EBF on their appearance, as they feared they would not look good enough for men. Most mothers in the current study were worried that they could lose their boyfriends because of the changes in their body image. The literature documents that young mothers want to avoid physical factors, such as sagging breasts, breast leakages, and breast milk-stained clothes to convince their boyfriends and peers that little had changed in their lives [52]. This problem is highly concerning, especially in a country where young mothers account for a substantial number of mothers, and EBF is important to improve child survival [57].

On the other hand, excessive loss of weight associated with EBF was a concern particularly among HIV negative mothers who feared that they would be perceived to be HIV positive and subjected to stigma. The findings are consistent with prior studies [55,56,57]. Discomfort about BF in public has the potential to negatively influence mothers who practice EBF, particularly those of low socioeconomic status who depend on public transport to move from one point to another. Discomfort about BF in public has been identified as a potential barrier to EBF in other studies in South Africa [29,52,58].

We further found that, consistent with other African societies [19,45], mixed feeding is a normal practice and has health impacts on the child’s health due to exposure to the risk of diarrhoea and malnutrition [8]. Mothers perceived breast milk to be inadequate and complained of the persistent crying of their babies, ultimately giving them solid food before time. Insufficient breastmilk has been associated with increased prevalence of mixed feeding early in the life of the infant in several studies in South Africa and other African settings [14,20,29]. The pressure exerted by the family to give the baby other food further exaggerates the problem of early introduction of solids and other liquids [14,19,29]. The need for concerted efforts to develop health-promotion messages geared towards the empowerment of mothers to protect EBF to the benefit of their exposed infants cannot be over-emphasised [45].

The reported benefits of BF expressed by mothers in this study are supported by the literature. The mothers in this study, as those in other studies, perceived BF to promote child growth and development, moreover that mothers perceived that their children were growing well as in other reports [59,60]. Furthermore, BF was perceived to promote mother–infant bonding, as well as to lower the risk of acquiring infectious diseases and the risk of child mortality [60]. Breastfeeding was also perceived as economical and readily available. The economical fact of BF indicated by mothers lies with the fact that buying formula is expensive. This is particularly important in this setting where women were of poor socioeconomic background in terms of prevalent single motherhood, low education level, unemployment, and low household income. UNICEF has tagged BF as one of the most effective and cost effective investments for the nations for current and future economic health [61], similar to the suggestion made in South Africa [62].

Prevention of child sickness through BF as reported by mothers in the current study is consistent to other reports that children who are not breastfed, or who are breastfed for short duration or at low intensity, have a higher risk of infection and illness than those who are breastfed optimally [63,64]. Ngoma-Hazemba and colleagues [45] argue that the perception that a breastfed baby is healthy is a practice that is rooted in the way of feeding babies in their culture. We also found that most of the women initiated BF because their mothers also breastfed them and their siblings. Evidence for a biologic link between BF and bonding is emerging, as BF mothers have higher brain responses to their own infants’ cry and exhibit more sensitive behaviour than formula-feeding mothers [65]. In the current study, mothers also related the benefits of BF to creating happiness and bonding with their children, as reported before [66]. Furthermore, there is evidence that mothers who breastfeed are likely to have improved health in the short-term and are at lower risk of developing future diseases [67,68,69]. Breastfeeding exclusively and for longer durations result in the most optimal maternal health [69].

## 5. Limitations of the Study

The strength of this study lies in using a qualitative approach, which enabled us to obtain rich data on mothers’ experiences on EBF during the first six months of the infant’s life and examine the extent to which initiation and sustenance of EBF is influenced by cultural beliefs and societal and family norms. Although we experienced challenges in forming FGDs and decided to do IDIs as well, this became another strength in integrating FGDs and IDIs, which enriched the interpretation of the findings and enhanced trustworthiness of findings. The 12 IDIs and three FGDs are typically sufficient to achieve data saturation [48,49], while 10 to 15 IDIs are suitable to answer a phenomenon studied [50]. Furthermore, we did not express the themes in frequency, meaning all emergent themes are equally important. This study had its own limitations, such as relying exclusively on mothers’ self-reports, which might have introduced recall and reporting bias, since it might have been difficult for some mothers to recall previous events regarding BF. Additionally, social desirability bias is acknowledged as a limitation since some mothers might have withheld information, and that cannot be overlooked.

## 6. Conclusions

The EBF experiences of mothers were rooted in cultural norms and traditional belief systems and conflicting beliefs on BF articulated as a personal practice, whilst these beliefs existed alongside fears of infecting the baby with HIV among HIV-positive mothers. The association of BF with sagging breasts and weight loss, as well discomfort with public BF, influenced initiation and early cessation of EBF. Shortened BF duration is highly concerning in relation to improving child survival.

EBF is recommended as the choice for infant feeding for HIV positive and negative mothers, and social norms, traditions, and cultural beliefs and practices influence mothers’ decision to initiate and sustain EBF. In settings where mixed feeding and ingestion of traditional concoctions are a norm, challenges remain on how to assist mothers to make an informed decision to choose and practice EBF.

It is therefore crucial that infant feeding messages ought to be context-specific to improve knowledge, understanding, acceptance and practice of EBF among HIV-positive and negative mothers. Culturally appropriate counselling messages that address the known cultural practices of the populations affected are essential to changing the beliefs and norms of the communities including extended families of EBF mothers.

## Figures and Tables

**Table 1:** Characteristics of mothers.

Variables	Categories/Mean ± SD	*n*	%
Age	27 ± 6		
	≤30 years	21	70
	>30 years	9	30
Marital status	Single	25	83
	Married	2	7
	Cohabiting	3	10
Education level	Primary	10	33
	Completed 12th Grade	18	60
	Tertiary	2	7
Employment status	Employed	8	27
	Unemployed	22	73
Household family income/month	<R2000 ($122.92)	21	70
	R2001–R5000 ($123.16–307.70)	8	27
	>R5000 ($307.70)	1	3
Pregnancy planned	No	20	67
	Yes	10	33
Attended ANC	No	4	13
	Yes	26	87
Time attended ANC	≤3 months	14	47
	>3 months	12	40
	Never attended	4	13
Maternal HIV status	Positive	12	40
	Negative	18	60
Delivery mode	Normal	21	70
	Caesarean section	9	30
Number of pregnancies	1	6	20
	2	9	30
	≥3	15	50
Parity	1	8	27
	2	8	27
	≥3	14	46

**Table 2:** Characteristics of children.

Variables	Categories/Mean ± SD	*n*	%
Child sex	9 ± 7		
	Boy	20	67
	Girl	10	33
Child age	<1 year	21	70
	>1 year	9	30
Childbirth order	Last	23	77
	Only	7	23
Child HIV status	Negative	30	100
	Positive	0	0

**Table 3:** Emergent themes from FGDs and IDIs.

Main Themes	Sub Themes
Child and maternal benefits of BF	BF promotes child growth and development
	BF offers protection against illness
	BF promotes mother-baby bonding
	BF is economical and convenient
	BF promotes maternal mental wellbeing
	Child spacing
Fears and distress of the effect of EBF	Fear of harming the baby
	Belief that the baby is not getting enough milk
	Fear of the effect of EBF on body image
Cultural beliefs influencing EBF	Personal practice
	Cultural and societal norms
	BF in public spaces
	Traditional beliefs

## Data Availability

The data that support the findings of this study are available from the corresponding author upon reasonable request.
